# ATP and AMP Mutually Influence Their Interaction with the ATP-binding Cassette (ABC) Adenylate Kinase Cystic Fibrosis Transmembrane Conductance Regulator (CFTR) at Separate Binding Sites* [Fn FN2]

**DOI:** 10.1074/jbc.M113.479675

**Published:** 2013-08-06

**Authors:** Christoph O. Randak, Qian Dong, Amanda R. Ver Heul, Adrian H. Elcock, Michael J. Welsh

**Affiliations:** From the Departments of ‡ Pediatrics,; § Biochemistry,; ¶ Internal Medicine, and; ‖ Molecular Physiology and Biophysics, University of Iowa, Iowa City, Iowa 52242 and; the ** Howard Hughes Medical Institute, Iowa City, Iowa 52242

**Keywords:** ABC Transporter, AMP, ATP, CFTR, Cystic fibrosis, Genetic Diseases

## Abstract

Cystic fibrosis transmembrane conductance regulator (CFTR) is an anion channel in the ATP-binding cassette (ABC) transporter protein family. In the presence of ATP and physiologically relevant concentrations of AMP, CFTR exhibits adenylate kinase activity (ATP + AMP ⇆ 2 ADP). Previous studies suggested that the interaction of nucleotide triphosphate with CFTR at ATP-binding site 2 is required for this activity. Two other ABC proteins, Rad50 and a structural maintenance of chromosome protein, also have adenylate kinase activity. All three ABC adenylate kinases bind and hydrolyze ATP in the absence of other nucleotides. However, little is known about how an ABC adenylate kinase interacts with ATP and AMP when both are present. Based on data from non-ABC adenylate kinases, we hypothesized that ATP and AMP mutually influence their interaction with CFTR at separate binding sites. We further hypothesized that only one of the two CFTR ATP-binding sites is involved in the adenylate kinase reaction. We found that 8-azidoadenosine 5′-triphosphate (8-N_3_-ATP) and 8-azidoadenosine 5′-monophosphate (8-N_3_-AMP) photolabeled separate sites in CFTR. Labeling of the AMP-binding site with 8-N_3_-AMP required the presence of ATP. Conversely, AMP enhanced photolabeling with 8-N_3_-ATP at ATP-binding site 2. The adenylate kinase active center probe P^1^,P^5^-di(adenosine-5′) pentaphosphate interacted simultaneously with an AMP-binding site and ATP-binding site 2. These results show that ATP and AMP interact with separate binding sites but mutually influence their interaction with the ABC adenylate kinase CFTR. They further indicate that the active center of the adenylate kinase comprises ATP-binding site 2.

## Teaser

**Background:** Cystic fibrosis transmembrane conductance regulator (CFTR) has adenylate kinase activity (ATP + AMP ⇆ 2 ADP).

**Results:** ATP enables CFTR photolabeling by 8-N_3_-AMP, and AMP increases 8-N_3_-ATP photolabeling at ATP-binding site 2.

**Conclusion:** AMP interacts with CFTR in an ATP-dependent manner and alters ATP interaction with the adenylate kinase active center ATP-binding site.

**Significance:** These findings exemplify nucleotide interactions with an ABC adenylate kinase.

## Introduction

**FIGURE 1.**
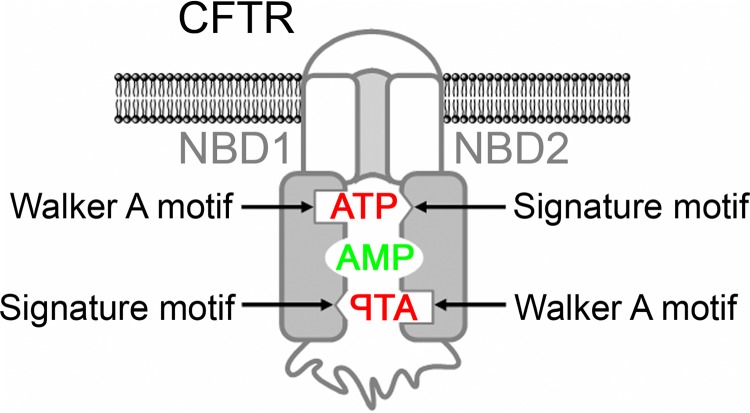
**Model of CFTR with separate binding sites for ATP and AMP.** ATP is sandwiched between the Walker A motif of one NBD and the ABC signature motif of the other NBD. The residues interacting with AMP are not known.

Cystic fibrosis transmembrane conductance regulator (CFTR)^3^ is an anion channel that belongs to the adenosine 5′-triphosphate (ATP)-binding cassette (ABC) family of proteins (1, 2). ABC proteins are defined by two highly conserved nucleotide-binding domains (NBDs) (3). The NBDs form a head-to-tail dimer that creates two ATP-binding sites (site 1 and site 2), each of which sandwiches ATP between the Walker A motif (phosphate-binding loop) (4) of one NBD and the ABC signature motif (5–7) of the other NBD (Fig. 1) (8–10).

ABC proteins can have two types of enzymatic activity, ATPase and adenylate kinase activity. ATPases hydrolyze ATP to adenosine 5′-diphosphate (ADP) and inorganic phosphate (P_i_) (ATP + H_2_O → ADP + P_i_), whereas adenylate kinases catalyze the transfer of the γ-phosphoryl group of ATP onto the α-phosphate of adenosine 5′-monophosphate (AMP) (ATP + AMP ⇆ 2 ADP). It is well known that CFTR opening and closing depends on ATPase activity when ATP is the only nucleotide present (11–14). In this case, ATP is bound to both ATP-binding sites and hydrolyzed at ATP-binding site 2 (15–18). However, patch clamp and biochemical studies showed that in the presence of physiologically relevant concentrations of AMP, adenylate kinase activity regulates CFTR channel function (19–22). The data further suggested that AMP interacted with a binding site distinct from the two ATP-binding sites and that ATP at ATP-binding site 2 was involved in the adenylate kinase activity (19, 20, 22). Both ATPase and adenylate kinase activity have been reported for two other ABC proteins, Rad50 (23) and SMC (structural maintenance of chromosome protein) (24).

The active center of an adenylate kinase comprises separate ATP- and AMP-binding sites (25–27). The first structural view of an ABC adenylate kinase became available when Lammens and Hopfner (24) solved the crystal structure of the NBD of the *Pyrococcus furiosus* SMC protein in complex with the adenylate kinase inhibitor P^1^,P^5^-di(adenosine-5′) pentaphosphate (Ap_5_A). Ap_5_A contains two adenosine groups connected by five phosphate groups, allowing it to bind simultaneously to the ATP- and the AMP-binding site (28, 29). These features make it a valuable experimental probe for an adenylate kinase active center. The SMC-NBD structure showed the two adenosine moieties of Ap_5_A attached to two binding sites separated by ∼15 Å. A Mg^2+^ ion, one adenosine, plus α-, β-, and γ-phosphates of Ap_5_A bound the canonical Mg^2+^-ATP-binding site on lobe I of the NBD. The other adenosine stacked onto the side chain of a conserved glutamine of the Q-loop at the interface of lobe I and lobe II. The authors also examined nucleotide-induced conformational changes. ATP did not alter the NBD structure. In contrast, binding of Ap_5_A rotated the two lobes of the NBD by ∼15°. These results suggest that adenylate kinase activity induces distinct conformational changes in SMC.

Although ATP and AMP bind separate sites, binding of one nucleotide influences the interaction with the other in non-ABC adenylate kinases. A number of different experimental approaches showed that ATP greatly increased binding of AMP to the AMP-binding site (30–33). Other studies demonstrated that binding of AMP to the AMP-binding site induced conformational changes in the ATP-binding domain (34–37).

Based on data from non-ABC adenylate kinases, we hypothesized that ATP and AMP mutually influence their interaction with CFTR at separate binding sites (Fig. 1). Based on the data from CFTR, we further hypothesized that only one of the two ATP-binding sites of CFTR is involved in adenylate kinase activity. To test these hypotheses, we employed the patch clamp technique with excised membrane patches containing CFTR and also photolabeling of CFTR using radioactive nucleotides with a photoactivatable azido (N_3_)-group attached to the adenine ring. The N_3_-group absorbs UV light, resulting in photolysis and formation of a reactive intermediate, which reacts covalently with nearby amino acid residues (38, 39). An advantage of these approaches is that they allow the interactions of nucleotides with CFTR to be studied while CFTR is embedded in the membrane.

## EXPERIMENTAL PROCEDURES

### 

#### 

##### Materials

The radioactive, photoactivatable azido-nucleotides, dissolved as triethylammonium salt in absolute methanol, were from Affinity Photoprobes, LLC (Lexington, KY). Immediately before use, the methanol was evaporated under a stream of argon, and the azido-nucleotide was dissolved in a buffer of 20 mm Hepes (pH 7.5), 50 mm NaCl, 3 mm MgCl_2_. Non-radioactive ATP, AMP, P^1^,P^4^-di(adenosine-5′) tetraphosphate (Ap_4_A), and Ap_5_A were from Sigma-Aldrich. ATP was used as magnesium salt. AMP and Ap_5_A were sodium salts, and Ap_4_A was an ammonium salt. The protease inhibitors used in this study were purchased from Sigma-Aldrich. Endoproteinase Arg-C from *Clostridium histolyticum* (sequencing grade) was from Roche Applied Science. Protein kinase A, catalytic subunit (PKA), purified from bovine heart, was from EMD Millipore Corp. (Billerica, MA). The monoclonal CFTR antibodies used were from R&D Systems, Inc. (Minneapolis, MN) (13-1 (40)), EMD Millipore (M3A7, L12B4 (41) and 13-4 (42)), and the Cystic Fibrosis Foundation in conjunction with the University of North Carolina (Chapel Hill, NC) (596 (42)).

##### Expression of CFTR in HeLa Cells and Preparation of Membranes

Wild-type and mutant CFTR were transiently expressed in HeLa cells using a double vaccinia virus/T7 RNA polymerase system (43). Cell membranes were prepared as described previously (22). The high speed membrane pellet (70,000 × *g*, 40 min, 4 °C) was resuspended in 20 mm Hepes (pH 7.5), 50 mm NaCl, 3 mm MgCl_2_, 2 μg/ml leupeptin, 100 μg/ml Pefabloc, and 7 μg/ml E-64.

##### CFTR Azido-nucleotide Photolabeling

Membranes (containing 25–35 μg of protein to detect photolabeling of full-length CFTR or 150–270 μg of protein to detect labeling of proteolytic CFTR fragments) were incubated on ice in 20 mm Hepes (pH 7.5), 50 mm NaCl, 3 mm MgCl_2_ with the radioactive azido-nucleotide and non-radioactive nucleotides as described in the figure legends in a total volume of 30 μl. Individual reactions were then irradiated with UV light (302 nm, 8-watt lamp) at a distance of 5 cm unless otherwise indicated. The duration of UV irradiation varied between the different azido-nucleotides and is indicated in the figure legends. After exposure to UV light, 20 μl of stop buffer (25 mm dithiothreitol, 4% SDS, 20 mm Hepes (pH 7.5), 50 mm NaCl, 125 μg/ml benzamidine, 4 μg/ml aprotinin, 2 μg/ml leupeptin, 100 μg/ml Pefabloc, 7 μg/ml E-64) followed by 875 μl of solubilization buffer (1% Triton X-100 in 20 mm Hepes (pH 7.5), 50 mm NaCl, 125 μg/ml benzamidine, 4 μg/ml aprotinin, 2 μg/ml leupeptin, 100 μg/ml Pefabloc, 7 μg/ml E-64) were added. Samples were stored at −80 °C overnight and thawed on ice before adding CFTR antibodies for immunoprecipitation. CFTR was immunoprecipitated as described (22) using monoclonal CFTR antibodies to its regulatory domain (13-1; 0.2 μg/sample) and NBD2 (M3A7; 1 μg/sample).

##### Partial Proteolysis with Arg-C

Immunocomplexed CFTR (from 150–270 μg of membrane protein) was bound to Dynabeads® Protein A (0.75 mg/sample) (Invitrogen). Beads were washed with 1 ml of 1% Triton X-100 in 20 mm Hepes (pH 7.5), 50 mm NaCl and 1 ml of 0.05% Triton X-100 in 20 mm Hepes (pH 7.5), 50 mm NaCl. The beads were then suspended in 18 μl of 82 mm Tris-HCl (pH 7.6), 9 mm CaCl_2_, 5 mm dithiothreitol, 0.75 mm EDTA, 0.09 μg of proteinase Arg-C and incubated for 2.5 h, gently shaking at 37 °C. The proteolysis reactions were stopped by adding dithiothreitol (end concentration 50 mm) and an equal volume of 2× Novex® Tricine SDS sample buffer (Invitrogen).

##### Gel Electrophoresis and Autoradiography

Immunoprecipitated full-length CFTR was fractionated on 6 or 8% SDS-polyacrylamide gels. Proteolytic digestion products of CFTR were fractionated on Novex® 16% Tricine gels (Invitrogen) (44). After electrophoresis, gels were either dried and then subjected to digital autoradiography using a FLA-7000 imaging system (Fuji Photo Film Co., Ltd., Tokyo, Japan) or transferred onto a PVDF membrane (Immobilon®-FL transfer membrane, EMD Millipore, Billerica, MA) for Western blotting. For quantitative image analysis, Multi Gauge analysis software (version 3.0; Fuji Photo Film) was used. Region intensities corresponding to protein or peptide bands were quantified in linear arbitrary units. Background intensities were subtracted.

##### Western Blotting

PVDF membranes blocked in 0.01% casein were incubated for 2 h with monoclonal anti-human CFTR antibodies as indicated for each experiment in the figures, diluted 1:1,000 in TTBS buffer (137 mm NaCl, 2.7 mm KCl, 25 mm Tris-Cl (pH 8.0), 0.05% Tween 20). Membranes were washed twice in TTBS buffer and then incubated for 1 h with donkey anti-mouse IRDye (0.1 μg/ml, in TTBS plus 0.01% casein, 0.01% SDS) (LI-COR Biosciences, Lincoln, NE) as secondary antibody. Immunoreactive proteins were visualized with the Odyssey infrared imaging system (LI-COR Biosciences).

##### Patch Clamp Experiments

CFTR Cl^−^ currents were studied using excised, inside-out membrane patches from HeLa cells transiently expressing either wild-type or mutant CFTR using a vaccinia virus/T7 RNA polymerase expression system (43) as described previously (20, 45). The pipette (extracellular) solution contained 140 mm *N*-methyl-d-glucamine, 2 mm MgCl_2_, 5 mm CaCl_2_, 100 mm l-aspartic acid, and 10 mm Tricine, pH 7.3, with HCl. The bath (intracellular) solution contained 140 mm *N*-methyl-d-glucamine, 3 mm MgCl_2_, 1 mm CsEGTA, and 10 mm Tricine, pH 7.3, with HCl. Following patch excision, CFTR channels were activated with 25 nm protein kinase A catalytic subunit (PKA) and ATP. PKA was present in all cytosolic solutions that contained ATP. Experiments were performed at room temperature (23–26 °C). Macropatch recordings were low pass-filtered at 100 Hz for analysis using an 8-pole Bessel filter (model 900, Frequency Devices, Inc. (Haverhill, MA)). Recordings from patches containing very few channels (A462F CFTR) with up to five simultaneous channel openings were low pass-filtered at 500 Hz for analysis. For these recordings, *NP_o_* was determined using the pCLAMP software package (version 9.2, Axon Instruments, Inc., Union City, CA).

##### Construction of a Three-dimensional Model of the NBD1-NBD2 Heterodimer

We constructed a putative molecular model of the human CFTR NBD1-NBD2 heterodimer in the following way. The crystal structure of NBD1 in complex with ATP (PDB code 1R0X) (46) was used as a template for constructing a homology model of NBD2 using the modeling program SWISSMODEL (47). Missing loop regions for NBD2, which were identified using the SEQATOMS Web server (48), were added using the loop-modeling program Loopy (49). Following the work of Callebaut *et al.* (50), a model of the CFTR NBD1-NBD2 heterodimer was assembled using the homodimeric structure of the MJ0796 ABC transporter from *Methanocaldococcus jannaschii* as a template (PDB code 1L2T) (9). Specifically, the NBD1-ATP crystal structure was superimposed onto chain A of the 1L2T structure using the “Iterative Magic Fit” tool in the modeling program Swiss-PdbViewer (51), with the Cα coordinates being used for the superposition. Similarly, the homology-modeled NBD2-ATP structure was superimposed onto chain B of the 1L2T structure. The resulting model of the NBD1-NBD2 heterodimer, together with the bound ATP molecules, is shown in Fig. 10.

##### Data Presentation and Statistics

Data are presented as means ± S.E. *p* values of <0.05 were considered statistically significant. For statistical analysis, SigmaStat software (version 3.0, SPSS Inc., Chicago, IL) was used.

## RESULTS

### 

#### 

##### ATP Enhances 8-N_3_-AMP Photolabeling and AMP Enhances 8-N_3_-ATP Photolabeling

**FIGURE 2.**
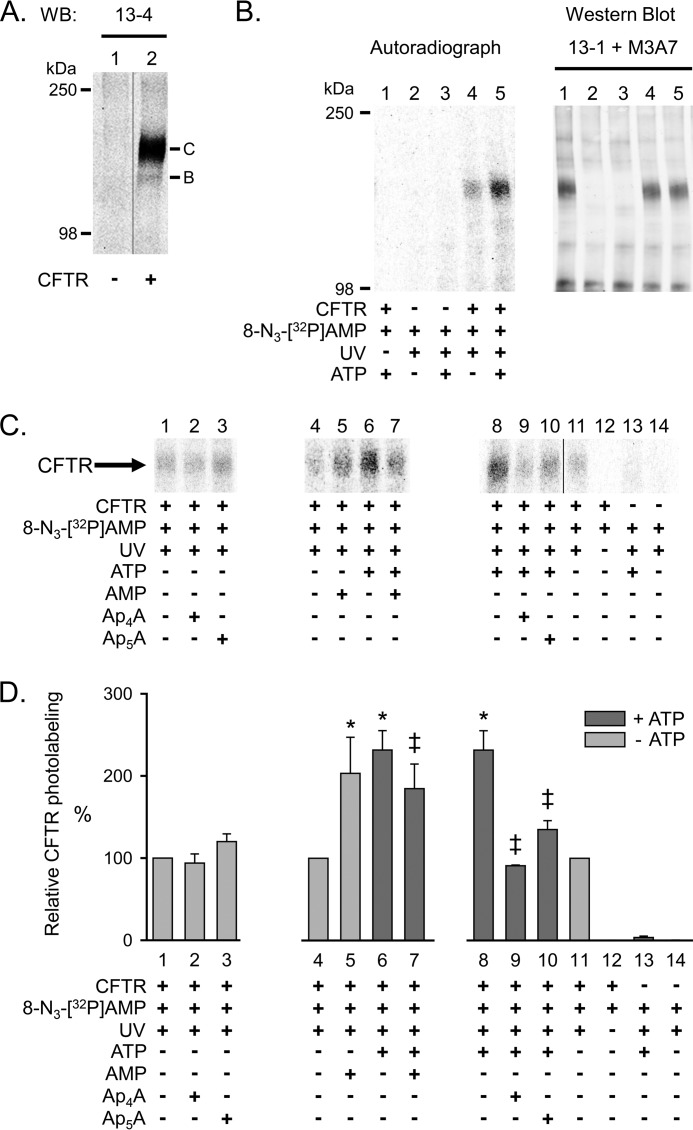
**Photolabeling of CFTR with 8-N_3_-[^32^P]AMP.** *A*, Western blot (*WB*) probed with CFTR antibody 13-4. *Letters* mark highly glycosylated (*C*) and core-glycosylated (*B*) CFTR. CFTR was immunoprecipitated from 30 μg of HeLa cell membrane protein as described under “Experimental Procedures.” No CFTR was detected in membranes from HeLa cells not infected with the recombinant vaccinia virus encoding CFTR (*lane 1*). *B*, autoradiograph (*left*) and Western blot (probed with CFTR antibodies 13-1 and M3A7) (*right*) of the same gel. Membranes were mixed on ice with 25 μm 8-N_3_-[^32^P]AMP in the absence and presence of 8.3 mm non-radioactive ATP as indicated *below* the *lanes* of the autoradiograph. The samples were immediately irradiated with UV light for 30 s. The sample of *lane 1* was not UV-irradiated. Comparing autoradiograph and Western blot corroborated that the labeled band was CFTR. *C*, autoradiographs from three different experiments labeling CFTR with 8-N_3_-[^32^P]AMP. Experimental conditions are indicated *below* the *lanes*. The concentration of non-radioactive ATP, AMP, Ap_4_A, and Ap_5_A was 8.3 mm. *D*, summary data. To compare the results from different autoradiographs, data were normalized to CFTR radioactivity under control conditions indicated *below bars 1*, *4*, and *11. Dark gray bars* mark labeling conditions in the presence of ATP. *, *p* = 0.012 (*bar 5*) and *p* < 0.001 (*bars 6* and *8*) compared with control (*bars 4* and *11*) (one-way repeated measures analysis of variance followed by Holm-Sidak's method of multiple comparisons *versus* control group, *n* = 2–10). ‡, *p* ≤ 0.015 compared with *bars 6* and *8* (one-way repeated measures analysis of variance followed by Holm-Sidak's method of multiple comparisons *versus* control group, *n* = 2–10). *Error bars*, S.E.

To test the hypothesis that ATP and AMP mutually influence their interaction with CFTR at separate binding sites, we expressed CFTR in HeLa cells using a double vaccinia virus/T7 RNA polymerase system (43) and collected cell membranes (22). Western blot analysis confirmed the presence of CFTR (Fig. 2*A*). The majority of the CFTR protein migrated as the highly glycosylated band C (40, 52). To investigate the interactions of ATP and AMP with CFTR, we used photolabeling with their azido (N_3_) nucleotide analogues. For ease of reading, we will refer to 8-N_3_-[^32^P]AMP as *AMP, and to 8-N_3_-[α-^32^P]ATP and 8-N_3_-[γ-^32^P]ATP as *ATP.

We tested the interaction of AMP with CFTR using *AMP. We detected photolabeling that increased in the presence of ATP (Fig. 2, compare *lanes 4* and *5* in *B*, *lanes 4* and *6* in C, and *lanes 8* and *11* in *C*). Photolabeling in the absence of ATP was not reduced by non-radioactive AMP (Fig. 2*C*, compare *lanes 4* and *5*). On the contrary, photolabeling increased in the presence of AMP, a phenomenon we will discuss below. Ap_4_A and Ap_5_A also failed to reduce labeling (Fig. 2*C*, compare *lane 1* with *lanes 2* and *3*). These data indicate that when *AMP is present alone, there is no specific photolabeling of an AMP-binding site. In the presence of ATP, non-radioactive AMP reduced photolabeling (Fig. 2*C*, compare *lanes 6* and *7*). Fig. 2*D* shows quantitative data. These results suggest specific labeling of an AMP-binding site in the presence of ATP. They further indicate that ATP- and AMP-binding sites are not identical.

**FIGURE 3.**
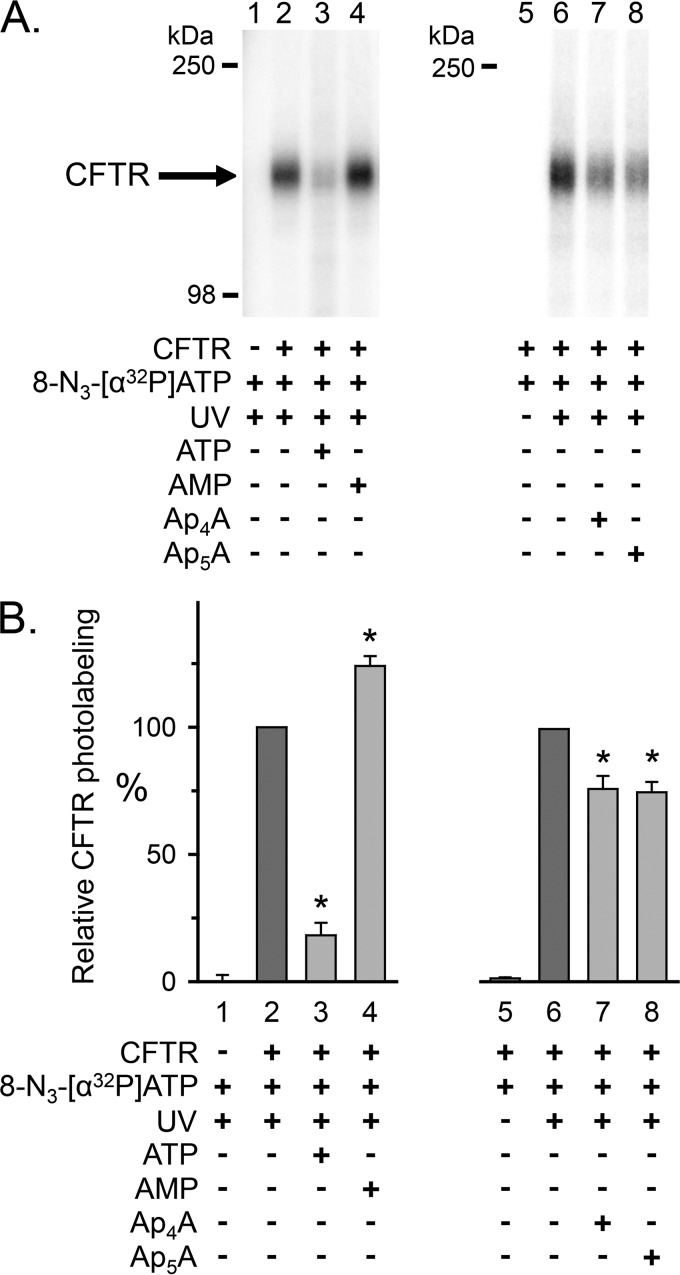
**Photolabeling of CFTR with 8-N_3_-[α-^32^P]ATP.** *A*, autoradiographs from two different experiments. Membranes were incubated on ice for 10 min with 50 μm 8-N_3_-[α-^32^P]ATP in the absence and presence of 5 mm non-radioactive ATP, AMP, Ap_4_A, or Ap_5_A as indicated *below* the *lanes* of the autoradiographs. The samples were subsequently irradiated with UV light for 90 s. The sample of *lane 5* was not UV-irradiated. CFTR was solubilized, immunoprecipitated, and fractionated on 8% (*lanes 1–4*) and 6% (*lanes 5–8*) SDS-polyacrylamide gels as described under “Experimental Procedures.” *B*, summary data. Experiments were performed as in *A*. Radioactivity incorporated into CFTR was normalized to radioactivity for the conditions indicated *below bars 2* and *6*. *, *p* ≤ 0.001 compared with control (*bars 2* and *6*) (one-way repeated measures ANOVA followed by Holm-Sidak's method of multiple comparisons *versus* control group, *n* = 6–7). *Error bars*, S.E.

To learn whether AMP influences the interaction of ATP with CFTR, we photolabeled with *ATP. Earlier studies showed that 8-N_3_-ATP binds both ATP sites of CFTR and, like ATP, supports CFTR channel activity (15–17, 53, 54). We also found that *ATP photolabeled CFTR, and non-radioactive ATP reduced labeling (Fig. 3*A*, compare *lanes 2* and *3*). Adding AMP consistently increased *ATP labeling by ∼20% (Fig. 3*A*, compare *lanes 2* and *4*). Fig. 3*B* shows quantitative data.

These data suggest that AMP interacts with CFTR in an ATP-dependent manner. They further suggest that AMP alters the interaction of ATP with CFTR.

##### Ap_5_A and Ap_4_A Inhibit 8-N_3_-AMP and 8-N_3_-ATP Photolabeling

As an additional test for interactions of AMP and ATP with sites that may be involved in adenylate kinase activity, we asked whether Ap_5_A and Ap_4_A would inhibit photolabeling by *AMP (in the presence of ATP) and *ATP. Ap_4_A, like Ap_5_A, is a double substrate adenylate kinase inhibitor that binds simultaneously to an ATP- and an AMP-binding site (PDB code 2C95).^4^ We previously showed that both Ap_4_A and Ap_5_A inhibit CFTR current (19). We found that both compounds inhibited *AMP labeling (Fig. 2*C*, compare *lanes 9* and *10 versus lane 8*). They also reduced photolabeling by *ATP (Fig. 3*A*, compare *lanes 7* and *8* with *lane 6*). These results are consistent with an interaction of Ap_5_A and Ap_4_A with both an AMP- and an ATP-binding site. However, Ap_5_A and Ap_4_A reduced labeling by *ATP to a lesser extent than did ATP, suggesting that they might not compete with *ATP at both ATP-binding sites.

**FIGURE 4.**
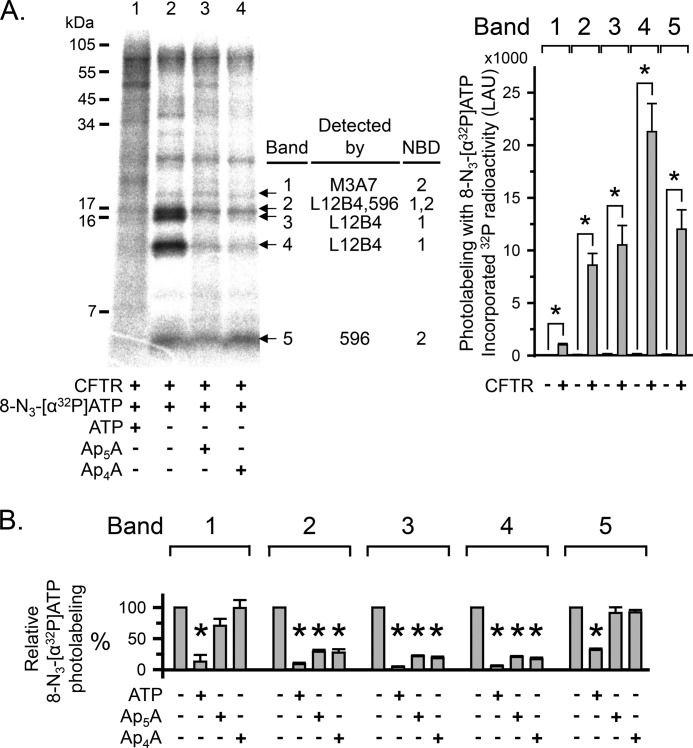
**Ap_5_A and Ap_4_A do not interact with all CFTR ATP-binding sites.** *A*, *left*, autoradiograph. Photolabeling of CFTR with 50 μm 8-N_3_-[α-^32^P]ATP was performed as described in the legend to Fig. 3. 5 mm non-radioactive ATP, Ap_4_A, or Ap_5_A were present as indicated *below* the *lanes* of the autoradiograph. After photolabeling, CFTR was solubilized, immunoprecipitated, and subjected to partial proteolysis with the proteinase Arg-C. The digestion products were fractionated on a 16% Tricine gel. To compare with undigested photolabeled and immunoprecipitated CFTR, see Fig. 3. *Middle*, summary of Western blot analysis results (see Fig. 5). *Right*, quantitative data in linear arbitrary units (*LAU*) for radioactivity incorporated into the different CFTR fragments (*bands 1–5*) after labeling with 8-N_3_-[α^32^P]ATP. *, *p* ≤ 0.024 compared with background control of no CFTR present (Mann-Whitney rank sum test, *n* = 3–8). *B*, quantitative data for photolabeling bands 1–5 with 50 μm 8-N_3_-[α-^32^P]ATP in the presence or absence of 5 mm non-radioactive ATP, Ap_4_A, or Ap_5_A. Amount of radioactivity incorporated into each band was normalized to radioactivity for control conditions (*i.e.* absence of non-radioactive ATP, Ap_4_A, or Ap_5_A). *, *p* ≤ 0.001 (one-way repeated measures ANOVA followed by Holm-Sidak's method of multiple comparisons *versus* control group, *n* = 2–5). *Error bars*, S.E.

**FIGURE 5.**
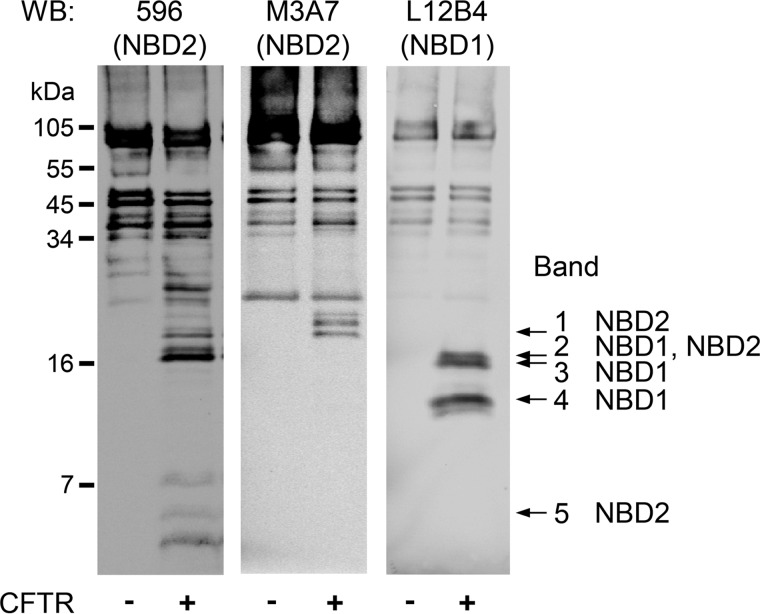
**Identification of CFTR NBD fragments produced by limited Arg-C digestion by Western blot analysis.** CFTR was solubilized, immunoprecipitated, and subjected to partial proteolysis with the proteinase Arg-C as described in the legend to Fig. 4. After fractionating on 16% Tricine gel, Western blots (*WB*) were probed with monoclonal antibodies to NBD1 (L12B4) and NBD2 (M3A7 and 596). These antibodies recognize epitopes within the following CFTR amino acid sequences: L12B4, 385–410; 596, 1204–1211; M3A7, 1373–1382 (42). *Bands 1–5* were identified by comparing autoradiographs and Western blots of the same gels.

To further assess this possibility, we labeled CFTR with *ATP, partially proteolyzed CFTR with Arg-C, and separated the digestion products on a 16% Tricine gel (Fig. 4*A*). We detected several labeled proteolytic CFTR fragments and focused on five small, labeled fragments (Fig. 4*A*, *bands 1–5*). We tested bands 1–5 with antibodies to NBD1 and NBD2 (Fig. 5). Bands 1 and 5 contained NBD2 sequence, whereas bands 3 and 4 contained NBD1 sequence. Band 2 was recognized by antibodies against NBD1 (L12B4) and NBD2 (596), suggesting that band 2 may represent two fragments of similar size originating from NBD1 and NBD2. Non-radioactive ATP reduced *ATP photolabeling of bands 1–5 (Fig. 4, *A* (compare *lanes 1* and *2*) and *B* (quantitative data)), indicating that the CFTR fragments of bands 1–5 interacted with or were in close proximity to ATP bound to ATP-binding site 1 or to ATP-binding site 2 in the intact CFTR protein. However, non-radioactive Ap_4_A and Ap_5_A only reduced labeling of bands 2, 3, and 4 (Fig. 4, *A* (compare *lanes 3* and *4* with *lane 2*) and *B* (quantitative data)). Thus, the binding sites for ATP and Ap_4_A/Ap_5_A overlapped, but there were more binding sites for ATP than for Ap_4_A and Ap_5_A. Because CFTR contains two ATP-binding sites, these results suggest that Ap_4_A and Ap_5_A interacted with one of the two ATP sites.

##### Ap_4_A and Ap_5_A Interact with an ATP- and an AMP-binding Site

To corroborate these results and to further characterize the interactions of Ap_4_A and Ap_5_A with CFTR, we used photolabeling with an N_3_-analog. Because a radioactive N_3_-Ap_5_A was not commercially available, we used 8-N_3_-[β_3_-^32^P]Ap_4_A. For ease of reading, we will refer to 8-N_3_-[β_3_^32^P]Ap_4_A as *Ap_4_A.

**FIGURE 6.**
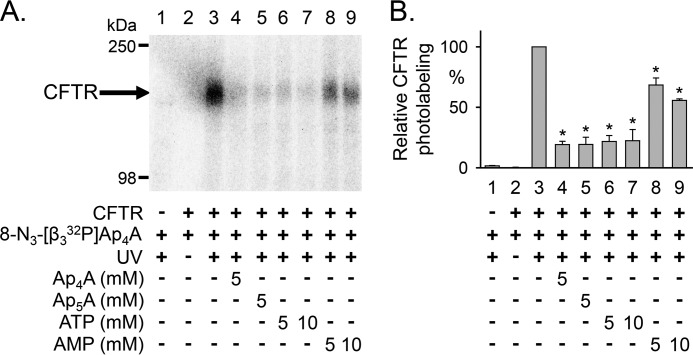
**Photolabeling of CFTR with 8-N_3_-[β_3_-^32^P]Ap_4_A.** *A*, autoradiograph. Membranes were mixed on ice with 25 μm 8-N_3_-[β_3_-^32^P]Ap_4_A in the absence and presence of non-radioactive ATP, AMP, Ap_4_A, or Ap_5_A, as indicated *below* the *lanes* of the autoradiograph. The samples were immediately irradiated with UV light for 30 s. *B*, summary data. Amount of radioactivity incorporated into CFTR was normalized to CFTR radioactivity under the conditions indicated *below bar 3*. *, *p* < 0.001 compared with *bar 3* (one-way repeated measures ANOVA followed by Holm-Sidak's method of multiple comparisons *versus* control group, *n* = 2–4). *Error bars*, S.E.

We found that *Ap_4_A readily labeled CFTR (Fig. 6*A*, *lane 3*). Labeling was UV light-dependent, and non-radioactive Ap_4_A and Ap_5_A reduced photolabeling. These results indicate that both Ap_4_A and Ap_5_A can interact with CFTR in the absence of other nucleotides. We also found that ATP and AMP reduced *Ap_4_A photolabeling. However, ATP interfered with *Ap_4_A photolabeling more effectively than AMP (Fig. 6*A*, *lane 3 versus lanes 6* and *7* compared with *lane 3 versus lanes 8* and *9*). Previous studies showed that ATP was also more effective than AMP at blocking *Ap_4_A labeling in chicken muscle adenylate kinase (56). These results suggest that when AMP is added in the absence of ATP, it interacts with CFTR with a lower affinity than ATP, Ap_4_A, and Ap_5_A.

**FIGURE 7.**
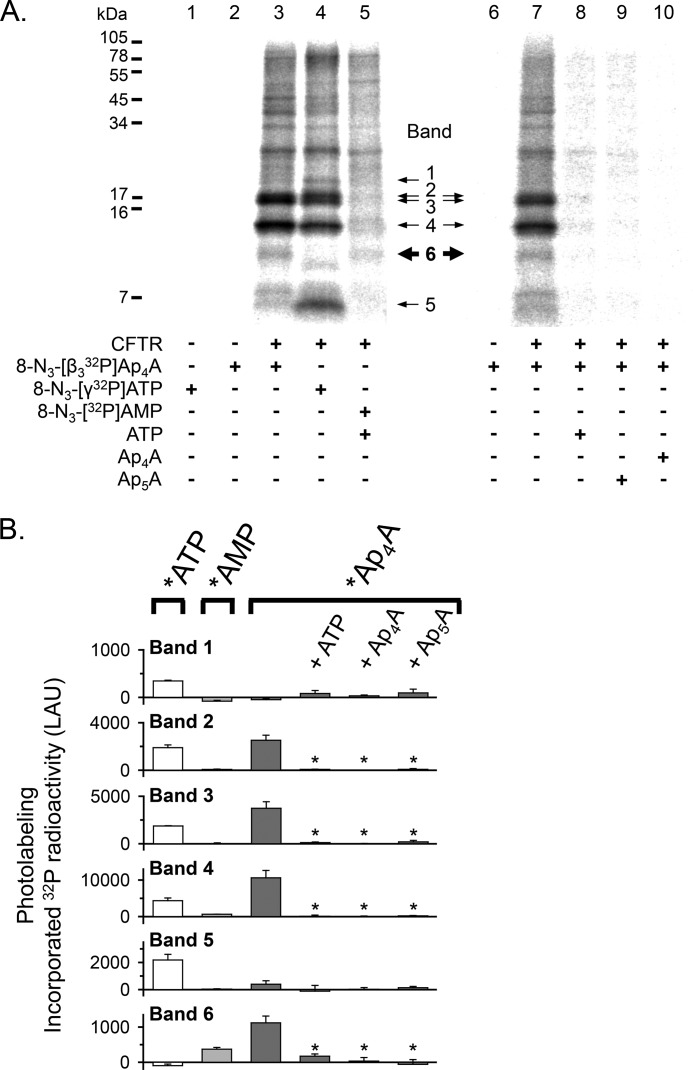
**Photolabeling of CFTR with 8-N_3_-[β_3_-^32^P]Ap_4_A, 8-N_3_-[γ-^32^P]ATP, and 8-N_3_-[^32^P]AMP.** *A*, autoradiographs from two different experiments. Photolabeling of CFTR was performed as described in the legend to Fig. 6. Non-radioactive ATP, Ap_4_A, or Ap_5_A was present in a concentration of 8.3 mm, as indicated *below* the *lanes* of the autoradiographs. In *lanes 1–5*, the three different radionucleotides, 8-N_3_-[β_3_-^32^P]Ap_4_A (10.3 Ci/mmol), 8-N_3_-[γ-^32^P]ATP (4.1 Ci/mmol), and 8-N_3_-[^32^P]AMP (5.8 Ci/mmol), were added at equal amounts of radioactivity (12.4 μCi). After photolabeling, CFTR was solubilized, immunoprecipitated, and subjected to partial proteolysis with the proteinase Arg-C. The digestion products were fractionated on a 16% Tricine gel. *B*, quantitative data in linear arbitrary units (*LAU*) for radioactivity incorporated into the different CFTR fragments after labeling with *Ap_4_A (*dark gray bars*), *ATP (*white bars*), and *AMP (*light gray bars*). *, *p* ≤ 0.005 compared with 8-N_3_-[β_3_-^32^P]Ap_4_A labeling under control conditions (*i.e.* absence of non-radioactive ATP, Ap_4_A, or Ap_5_A (*dark gray bars*)) (one-way repeated measures ANOVA followed by Holm-Sidak's method of multiple comparisons *versus* control group, *n* = 3–5). *Error bars*, S.E.

To test the hypothesis that *Ap_4_A labeled both an ATP-binding site and a separate AMP-binding site, we compared the proteolytic fragments labeled by *ATP and *AMP with those labeled by *Ap_4_A. We found the following. (*a*) Again, *ATP labeled bands 1–5 (Fig. 7, *A* (*lane 4*) and *B* (*white bars*)). (*b*) The labeling pattern with *AMP was different from that with *ATP (Fig. 7, *A* (compare *lanes 4* and *5*) and *B* (compare *white* and *light gray bars*)). *AMP photolabeled a proteolytic fragment of ∼10 kDa (Fig. 7, *A* and *B*, *band 6*) that was not labeled by *ATP. Note that as described above, non-radioactive ATP was present when band 6 was labeled by *AMP (Fig. 7*A*, *lane 5*). This fragment was not detected in the absence of CFTR. It was not recognized by the NBD antibodies L12B4, M3A7, and 596 that reacted with fragments 1–5 (Fig. 5), presumably because it did not contain the epitope for those antibodies. These data suggest that CFTR contains a binding site for AMP that is distinct from the ATP-binding sites. (*c*) *Ap_4_A photolabeled a subset of fragments marked by *ATP (*bands 2–4*; Fig. 7, *A* (compare *lanes 3* and *4*) and *B* (compare *white* and *dark gray bars*)), consistent with *Ap_4_A labeling only one of the two ATP-binding sites. In addition, like *AMP, *Ap_4_A labeled band 6 (Fig. 7, *A* (compare *lanes 3* and *5*) and *B* (compare *light* and *dark gray bars*)). These data suggest that *Ap_4_A labels both an ATP- and an AMP-binding site. (*d*) ATP, as well as Ap_4_A and Ap_5_A, prevented labeling by *Ap_4_A (Fig. 7, *A* (compare *lanes 8–10* with *lane 7*) and *B* (*dark gray bars*)). Interestingly, non-radioactive ATP prevented photolabeling of band 6 by *Ap_4_A, although *ATP did not label band 6. These data suggest that photolabeling of band 6 by *Ap_4_A required that *Ap_4_A also interacted with an ATP-binding site at the time of labeling. This result is consistent with the finding that ATP facilitated *AMP labeling (Fig. 2).

##### AMP Enhances 8-N_3_-ATP Photolabeling at the Site Labeled by 8-N_3_-Ap_4_A

**FIGURE 8.**
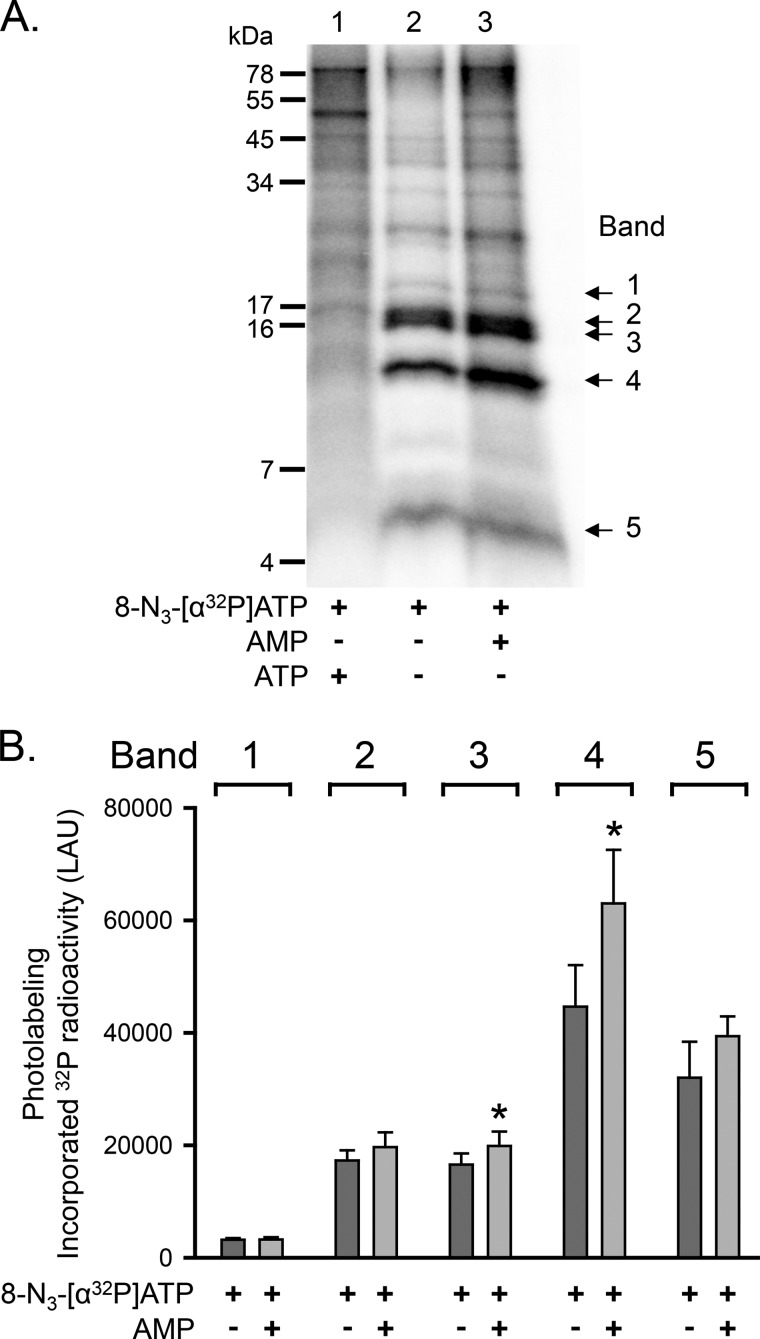
**Increased photolabeling of CFTR with 8-N_3_-[α-^32^P]ATP at one ATP-binding site in the presence of AMP.** *A*, autoradiograph. Experiments were performed as described in the legend to Fig. 4. The concentration of non-radioactive ATP and AMP was 5 mm. *B*, quantitative data in linear arbitrary units (*LAU*) for radioactivity incorporated into bands 1–5 after labeling with 8-N_3_-[α-^32^P]ATP. The absence (*dark gray bars*) or presence of AMP (*light gray bars*) is indicated *below* each *bar*. *, *p* ≤ 0.016 (paired *t* test, *n* = 4). *Error bars*, S.E.

Ap_5_A inhibits adenylate kinase activity by binding to the enzyme's active center (*i.e.* the phosphate donor ATP-binding site and the phosphate acceptor AMP-binding site) (26, 29). We hypothesized that the increase in CFTR photolabeling with *ATP in the presence of AMP (Fig. 3) involved the same ATP-binding site that interacted with Ap_4_A/Ap_5_A. As shown above, *Ap_4_A labeled a subset of the proteolytic fragments that were labeled by *ATP (*i.e.* bands 2–4) (Fig. 7), and Ap_5_A reduced *ATP photolabeling of bands 2–4 but not of bands 1 and 5 (Fig. 4). We therefore predicted that AMP would enhance *ATP labeling of band 2, 3, or 4. Consistent with that prediction, AMP mainly increased *ATP photolabeling of band 4 as well as, to a small extent, of band 3, whereas labeling of bands 1, 2, and 5 did not significantly change (Fig. 8*A*, compare *lanes 2* and *3*). Fig. 8*B* shows quantitative data. Although this assay has the limitation that it may not detect differences smaller than 25%, the results indicate that AMP enhances *ATP photolabeling at the same ATP-binding site that interacts with Ap_4_A/Ap_5_A (*i.e.* that interacts with the phosphate donor ATP).

##### Ap_5_A Interacts with ATP-binding Site 2

**FIGURE 9.**
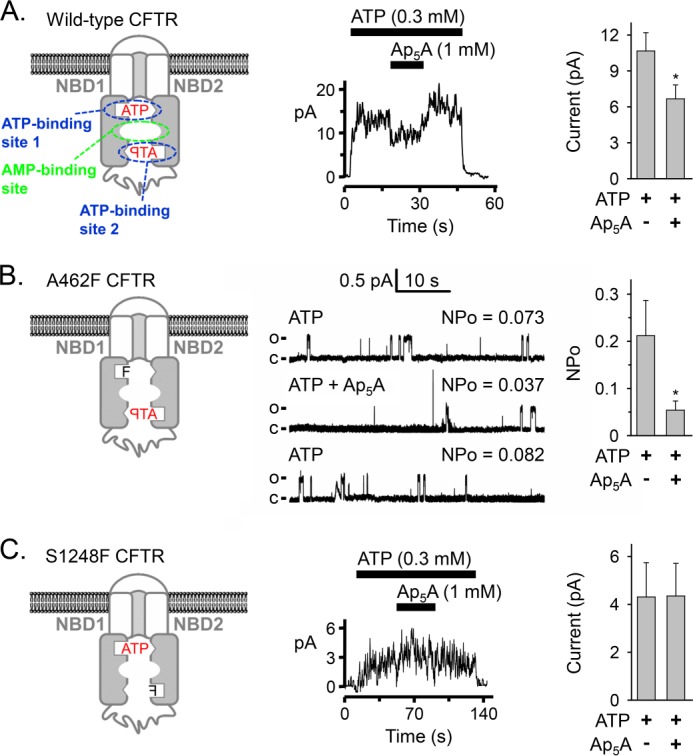
**Effect of phenylalanine mutations in the phosphate-binding loop (Walker A motif) of either ATP-binding site on Ap_5_A inhibition of CFTR current.** *A*, *left*, model of CFTR. The phosphate-binding loops are depicted as *open rectangles*, and the ABC signature motifs are shown as *open triangles*. The binding site for AMP is not known. *Middle*, current recording (100 ms averages) from an excised inside-out membrane patch containing multiple CFTR channels. ATP and Ap_5_A were present during the times and at the concentrations indicated by *bars*. ATP was added together with PKA catalytic subunit. Holding voltage was −50 mV. *Right*, CFTR Cl^−^ current before and after adding 1 mm Ap_5_A. Experiments were performed as shown in the *middle panel* with 0.3 mm ATP and PKA present. *, *p* < 0.001 (Wilcoxon signed rank test, *n* = 13 paired experiments of current measurements before and after adding Ap_5_A obtained from five membrane patches). *B*, *left*, model of A462F CFTR. *Middle*, current recording from one excised inside-out membrane patch containing at least two A462F CFTR channels perfused on cytosolic surface with ATP and Ap_5_A as indicated. PKA catalytic subunit was present throughout the recording. Holding voltage was −50 mV. Each *lane* shows 48 s of recording and mean *NP_o_*. The *first lane* shows the trace in the presence of 0.3 mm ATP, the *second lane* shows the recording after 1 mm Ap_5_A was added, and the *third lane* shows the recording after Ap_5_A was removed again. For illustration purposes, traces were digitally low pass-filtered at 50 Hz. *c*, channel closed state; *o*, single channel open state. *Right*, *NP_o_* of A462F CFTR with 0.3 mm ATP and PKA present in the bath solution before and after adding 1 mm Ap_5_A. *, *p* = 0.002 (Wilcoxon signed rank test, *n* = 10 membrane patches). *C*, *left*, model of S1248F CFTR. This mutant contained an N-terminal 6-histidine tag between CFTR amino acids 2 and 3. *Middle*, current recording (100 ms averages) from an excised inside-out membrane patch containing multiple S1248F CFTR channels. ATP and Ap_5_A were present during the times and at the concentrations indicated by *bars*. ATP was added together with PKA catalytic subunit. Holding voltage was −80 mV. *Right*, S1248F CFTR Cl^−^ current before and after adding 1 mm Ap_5_A. Experiments were performed as shown in the *middle panel* with 0.3 mm ATP and PKA present. No significant differences were detected (*p* = 0.463, Wilcoxon signed rank test, *n* = 14 paired experiments of current measurements before and after adding Ap_5_A obtained from four membrane patches). *Error bars*, S.E.

As previously shown, Ap_5_A inhibited wild-type CFTR Cl^−^ current (19, 20, 57) (Fig. 9*A*, *middle* and *right*). To learn whether inhibition was due to an interaction with ATP-binding site 1 or site 2, we studied the effect of phenylalanine substitutions in the phosphate-binding loops (Walker A motifs). ATP-binding site 1 contains the phosphate-binding loop of NBD1, whereas ATP-binding site 2 contains the phosphate-binding loop of NBD2 (Fig. 9*A*, *left*). Berger *et al.* (15) found that substituting alanine at position 462 in NBD1 with phenylalanine (A462F mutation; Fig. 9*B*, *left*) abolished nucleotide interaction with ATP-binding site 1. Substituting serine at position 1248 in NBD2 with phenylalanine (S1248F mutation; Fig. 9*C*, *left*) abolished nucleotide interaction with ATP-binding site 2. Both mutants exhibited a low, ATP-dependent open probability due to a reduced opening rate with a normal burst duration (15). The A462F, but not the S1248F mutation interfered with processing and trafficking to the cell membrane (supplemental Fig. S1), and hence, the number of channels in excised membrane patches was small; therefore, we quantified channel activity as *NP_o_*. We found that Ap_5_A reduced the *NP_o_* of A462F CFTR (Fig. 9*B*, *middle* and *right*). On the other hand, Ap_5_A had no effect on ATP-dependent current of S1248F CFTR (Fig. 9*C*, *middle* and *right*). Thus, preventing the interaction of nucleotides with ATP-binding site 2 eliminated Ap_5_A inhibition. Our results are consistent with the previous observations that mutations of conserved residues in the Walker A and B motifs of ATP-binding site 2, K1250A and D1370N, abolished Ap_5_A inhibition of current, whereas the homologous mutations in ATP-binding site 1, K464A and D572N, did not. These results indicate that Ap_5_A interacts with ATP-binding site 2 and not site 1 to inhibit current.

## DISCUSSION

Our data indicate that ATP and AMP interact with distinct sites in CFTR, and they mutually influence their interactions. Furthermore, our patch clamp data suggest that the adenylate kinase active center contains ATP-binding site 2.

We found that *AMP photolabeled CFTR in an ATP-dependent manner. ATP may have assisted AMP binding or azido-group cross-linking. This effect may require ATP to interact with both ATP-binding sites or with only one of them. Of note, a number of approaches have demonstrated an ATP-dependent interaction with AMP in soluble, non-ABC adenylate kinases. (*a*) Several studies indicate that ATP increases binding of AMP to the AMP-binding site. Studies with beef heart mitochondrial adenylate kinase and a closely related GTP:AMP phosphotransferase showed that AMP bound to the enzyme only in the presence of nucleotide triphosphate (30, 31). (*b*) The Michaelis constant (*K_m_*) for AMP of bakers' yeast adenylate kinase is significantly higher if ATP is substituted by GTP. GTP binds with lower affinity than ATP and generates a lower catalytic rate (33), indicating that substrate binding at the nucleotide triphosphate binding site influenced the interaction of AMP at the AMP-binding site. Furthermore, mutations within the ATP-binding site that increase *K_m_* for ATP also increase *K_m_* for AMP (29).

In addition, we observed some augmentation of *AMP photolabeling when non-radioactive AMP was added in the absence of ATP (Fig. 2*C*, compare *lanes 4* and *5*). We speculate that AMP might bind the ATP-binding site under these conditions, which may increase *AMP labeling at the AMP-binding site(s). This speculation is founded on results with bakers' yeast and porcine muscle adenylate kinase showing that (*a*) ATP facilitated AMP binding, and (*b*) in the absence of ATP, AMP bound preferentially to the ATP-binding site although with much lower affinity than ATP (32, 33, 58).

Our experiments also showed that AMP increased CFTR photolabeling by *ATP. The data suggest that *ATP photolabeling increased at the same ATP-binding site that binds Ap_5_A. We speculate that an AMP-induced conformational change might be responsible (*i.e.* one similar to that described for SMC) (24). These biochemical data complement previous electrophysiological data: patch clamp experiments had shown that AMP induced positive cooperativity into the relationship between ATP concentration and current, indicating that AMP affected the interaction of ATP with CFTR. AMP-induced conformational changes affecting the interaction of ATP with the enzyme have been proposed from structural data or experimentally demonstrated for other adenylate kinases (34–37). For example, in a study using an ATP analog with an environment-sensitive fluorescent group to probe the ATP-binding site of rabbit muscle adenylate kinase, Chuan *et al.* (35) showed that fluorescence increased in the presence of AMP. This result indicated that the environment of the fluorescent probe at the ATP-binding site changed when AMP was added, suggesting a conformational change (35).

Our results specify that the adenylate kinase inhibitor Ap_5_A interacts with only one of the two ATP-binding sites of CFTR, ATP-binding site 2. Because Ap_5_A binds to the active center of adenylate kinases, these data suggest that ATP-binding site 2 interacts with the phosphoryl group donor ATP. Previous studies are consistent with this conclusion. (*a*) The S1248F mutation, which interfered with the binding of ATP to site 2 (15) and abolished the inhibition of CFTR current in the presence of Ap_5_A in our study (Fig. 9*C*), also disrupted CFTR adenylate kinase activity (22). (*b*) Patch clamp studies showed that CFTR mutations K1250A and D1370N, located within the conserved Walker A and B motifs of ATP-binding site 2, abolished the effects of Ap_5_A and AMP on CFTR current. However, the homologous mutations in ATP-binding site 1 (K464A and D572N) did not (19). Our data plus those observations indicate that adenylate kinase activity involves the same ATP-binding site at which ATP is hydrolyzed in the absence of AMP (15–18).

In current models of CFTR, both ATP molecules are bound between the phosphate-binding loop (Walker A motif) of one NBD and the ABC signature motif of the other NBD (Fig. 9*A*, *left*) (50, 59, 60). Thus, both NBDs contribute to both binding sites. These models are based on the crystal structures of other ABC proteins and corroborated by patch clamp data suggesting ATP-driven NBD1-NBD2 dimerization opening the CFTR channel pore (61). Our patch clamp results suggest that Ap_5_A interacts with the phosphate-binding loop (Walker A motif) of NBD2 but not with the phosphate-binding loop of NBD1. In addition, we showed that Ap_5_A prevented *ATP photolabeling of CFTR proteolytic fragments containing NBD1 sequences (*bands 2–4* in Figs. 4 and 5). *ATP photolabeling occurs at protein sequences in close proximity to the N_3_-group at the adenine ring. These results are consistent with Ap_5_A and ATP binding at the interface of an NBD1-NBD2 dimer.

**FIGURE 10.**
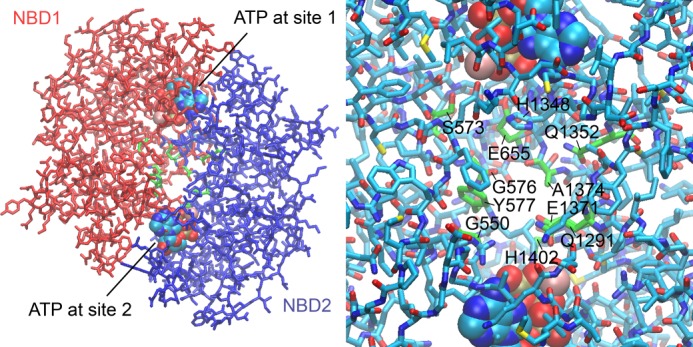
**Three-dimensional model of the CFTR NBD1-NBD2 heterodimer.** The model was constructed as described under “Experimental Procedures.” *Left*, *stick model* of the overall heterodimer structure with NBD1 in *red* and NBD2 in *blue*. Two ATP molecules (in a *space-filling representation*) are bound between the Walker A motif of one NBD and the ABC signature motif of the other NBD. A central space between the two NBDs is evident, into which one adenosine moiety of Ap_5_A could extend. Residues lining this space from both NBDs are depicted in *green. Right*, *close-up view* of the central space region between the two NBDs. The residues in *green* lining the cavity might interact with AMP. Histidine 1348 might prevent Ap_5_A from interacting with ATP-binding site 1. See supplemental Movie S1 to facilitate visualizing the tree-dimensional positioning of these residues.

To speculate about the structural basis of our findings, we constructed a three-dimensional homology model of the CFTR NBD1-NBD2 heterodimer (Fig. 10 and supplemental Movie S1). The model revealed a central cavity formed by residues from both NBD1 and NBD2, particularly Gly-550, Ser-573, Gly-576, Tyr-577, Glu-655, Gln-1291, Gln-1352, Glu-1371, Ala-1374, and His-1402. If one adenosine moiety of Ap_5_A were bound to ATP-binding site 2, the other adenosine, the “AMP” adenosine, could extend into the central cavity. Although the conformation of the bound Ap_5_A cannot be predicted, it would probably interact with a subset of the residues lining the cavity. The same residues would be expected to interact with AMP. Of note, Gln-1291 is the conserved Q-loop glutamine of NBD2 (8, 62). In the crystal structure of an SMC-NBD, the “AMP” adenosine of Ap_5_A is bound to the SMC Q-loop glutamine (24). Interestingly, mutations of several residues lining the central cavity have been observed in human disease (see the Cystic Fibrosis Mutation Database, Cystic Fibrosis Centre at the Hospital for Sick Children, Toronto, Canada). Mutation Q1291R has been found in patients with cystic fibrosis. Mutations G576A, G550R, and Q1352H have been described in patients with congenital bilateral absence of the vas deferens, a condition that affects men with cystic fibrosis but can also occur in the absence of other disease manifestations. Because in our model, both NBDs contribute to the central cavity, the interaction of AMP with CFTR is predicted to be influenced by NBD1-NBD2 dimerization, which is linked to ATP binding. Our finding that *AMP photolabeled CFTR in an ATP-dependent manner is consistent with this model. Our model shows His-1348 in a position where it might prevent Ap_5_A from interacting simultaneously with ATP-binding site 1 and central cavity residues. We speculate that this structure may explain our results indicating that Ap_5_A interacts only with ATP-binding site 2.

Previous work showed that Ap_5_A partially inhibits wild-type CFTR channel activity (19, 57). In the case of CFTR mutants, the interaction with Ap_5_A may increase current. An example is L1254A CFTR, which showed an increase in current after adding Ap_5_A due to a reduced channel closing rate. The mutation is predicted to affect the interaction of ATP and Ap_5_A at ATP-binding site 2 (45). Although Ap_5_A itself is not cell-permeable and is not therefore a potential pharmaceutical, our studies may lead to the development of therapeutic strategies to positively or negatively modulate the channel activity of CFTR. Those may be of potential value for the treatment of diseases associated with decreased (cystic fibrosis) or increased (cholera toxin-induced secretory diarrhea (55)) CFTR chloride currents.

## Supplementary Material

Supplemental Data

## Footnotes

4 G. Bunkoczi, P. Filippakopoulos, A. Jansson, E. Longman, F. Von Delft, A. Edwards, C. Arrowsmith, M. Sundstrom, J. Weigelt, and S. Knapp, unpublished results.

## Footnotes

3 The abbreviations used are:

CFTR: cystic fibrosis transmembrane conductance regulator
ABC: ATP-binding cassette
8-N_3_-ATP: 8-azidoadenosine 5′-triphosphate
8-N_3_-AMP: 8-azidoadenosine 5′-monophosphate
8-N_3_-[^32^P]AMP: 8-[^32^P]azidoadenosine 5′-monophosphate
8-N_3_-[β_3_-^32^P]Ap_4_A: [β_3_-^32^P]P^1^-(adenosyl-5′)-P^4^-(8-azido-adenosyl-5′)tetraphosphate
8-N_3_-[α-^32^P]ATP: 8-[α-^32^P]azidoadenosine 5′-triphosphate
8-N_3_-[γ-^32^P]ATP: 8-[γ-^32^P]azidoadenosine 5′-triphosphate
*AMP: 8-N_3_-[^32^P]AMP
*Ap_4_A: 8-N_3_-[β_3_-^32^P]Ap_4_A
*ATP: 8-N_3_-[^32^P]ATP
Ap_4_A: P^1^,P^4^-di(adenosine-5′) tetraphosphate
Ap_5_A: P^1^,P^5^-di(adenosine-5′) pentaphosphate
NBD: nucleotide-binding domain
SMC: structural maintenance of chromosome
Tricine: *N*-[2-hydroxy-1,1-bis(hydroxymethyl)ethyl]glycine
E-64: *trans*-epoxysuccinyl-l-leucylamido-(4-guanidino)butane
ANOVA: analysis of variance
PDB: Protein Data Bank.
