# Supplementary material for: ATP and AMP Mutually Influence Their Interaction with the ATP-binding Cassette (ABC) Adenylate Kinase Cystic Fibrosis Transmembrane Conductance Regulator (CFTR) at Separate Binding Sites
Source: J Biol Chem. 2013 Aug 6;288(38):27692–701. doi: 10.1074/jbc.M113.479675 (PMC3779764; doi:10.1074/jbc.M113.479675)
Supplement: Supplemental Data [file supp_288_38_27692__index.html]

ATP and AMP Mutually Influence Their Interaction with the ATP-binding Cassette (ABC) Adenylate Kinase Cystic Fibrosis Transmembrane Conductance Regulator (CFTR) at Separate Binding Sites — Nucleotide Interactions with the ABC Adenylate Kinase CFTR — Supplemental Data 

# ATP and AMP Mutually Influence Their Interaction with the ATP-binding Cassette (ABC) Adenylate Kinase Cystic Fibrosis Transmembrane Conductance Regulator (CFTR) at Separate Binding Sites

## Supplemental Data

**Files in this Data Supplement:**

- Supplemental Figure S1 - Processing of CFTR with phenylalanine substitutions in the Walker A motifs
- Supplemental Movie S1  - Central cavity in the three-dimensional model of the CFTR NBD1-NBD2 heterodimer
