# Supplementary material for: ATP and AMP Mutually Influence Their Interaction with the ATP-binding Cassette (ABC) Adenylate Kinase Cystic Fibrosis Transmembrane Conductance Regulator (CFTR) at Separate Binding Sites
Source: J Biol Chem. 2013 Aug 6;288(38):27692–701. doi: 10.1074/jbc.M113.479675 (PMC3779764; doi:10.1074/jbc.M113.479675)
Supplement: Supplemental Data [file supp_M113.479675_jbc.M113.479675-1.pdf]

SUPPLEMENTAL DATA

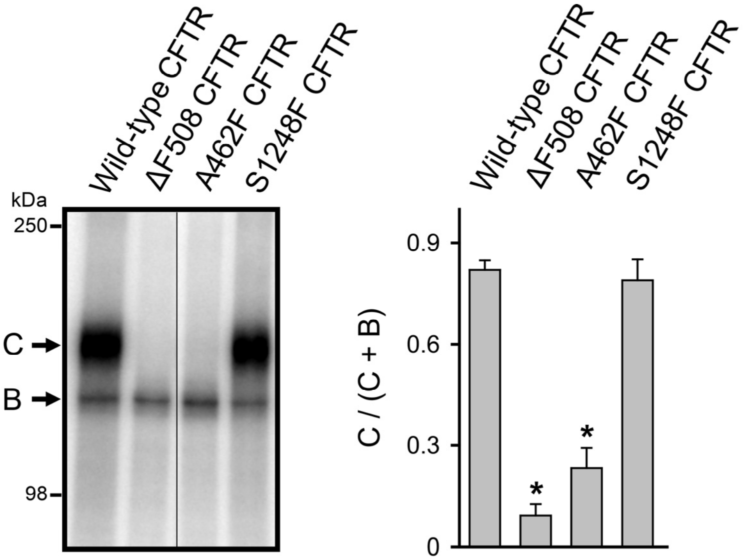

FIGURE S1. Processing of CFTR with phenylalanine substitutions in the Walker A motifs. Processing was evaluated by assessing CFTR glycosylation. CFTR receives core glycosylation in the endoplasmic reticulum. The core glycosylated CFTR migrates as "band B" during SDS gel electrophoresis. CFTR becomes highly glycosylated in the Golgi apparatus and then migrates as "band C". Deletion of phenylalanine 508 (ΔF508) exemplifies a mutation that causes a processing defect at 37°C: the majority of mutant protein is degraded before reaching the Golgi complex and therefore does not migrate as the fully glycosylated band C (1-4). **A.** Autoradiograph. Wild-type and mutant CFTR were transiently expressed in 293T cells for 48 hours at 37°C using a vaccinia virus-T7 hybrid expression system (5). CFTR was solubilized, immunoprecipitated and phosphorylated with the catalytic subunit of protein kinase A and [γ^{32}P]ATP (6). Immunoprecipitates were fractionated on 6% SDS polyacrylamide gels as described in the methods section. **B.** Quantitative data for the fraction of CFTR migrating as band C. Radioactivity incorporated into bands B and C was quantified by digital autoradiography as described in the methods section. Depicted is the ratio of radioactivity in band C versus the total radioactivity in bands B and C. Asterisks indicate $p<0.05$ compared to wild-type CFTR (Kruskal-Wallis One Way ANOVA on Ranks followed by Dunn's method of multiple comparisons versus control group; wild-type CFTR, n = 13; ΔF508 CFTR, n = 10; A462F CFTR, n = 4; S1248F CFTR, n = 4). The fraction of CFTR protein with a phenylalanine substitution at position 462 (A462F) migrating as band C is significantly lower than that of wild-type CFTR.

References:

1. Gregory, R. J., Cheng, S. H., Rich, D. P., Marshall, J., Paul, S., Hehir, K., Ostedgaard, L., Klinger, K. W., Welsh, M. J., and Smith, A. E. (1990) Expression and characterization of the cystic fibrosis transmembrane conductance regulator. *Nature* **347**, 382-386
2. Cheng, S. H., Gregory, R. J., Marshall, J., Paul, S., Souza, D. W., White, G. A., O'Riordan, C. R., and Smith, A. E. (1990) Defective intracellular transport and processing of CFTR is the molecular basis of most cystic fibrosis. *Cell* **63**, 827-834
3. Lukacs, G. L., Mohamed, A., Kartner, N., Chang, X. B., Riordan, J. R., and Grinstein, S. (1994) Conformational maturation of CFTR but not its mutant counterpart (ΔF508) occurs in the endoplasmic reticulum and requires ATP. *EMBO J.* **13**, 6076-6086
4. Ward, C. L., and Kopito, R. R. (1994) Intracellular turnover of cystic fibrosis transmembrane conductance regulator. Inefficient processing and rapid degradation of wild-type and mutant proteins. *J. Biol. Chem.* **269**, 25710-25718
5. Ostedgaard, L. S., and Welsh, M. J. (1992) Partial purification of the cystic fibrosis transmembrane conductance regulator. *J. Biol. Chem.* **267**, 26142-26149
6. Dong, Q., Ostedgaard, L. S., Rogers, C., Vermeer, D. W., Zhang, Y., and Welsh, M. J. (2012) Human-mouse cystic fibrosis transmembrane conductance regulator (CFTR) chimeras identify regions that partially rescue CFTR-DeltaF508 processing and alter its gating defect. *Proc Natl Acad Sci U S A* **109**, 917-922
